# Interfacial Ca^2+^ environments in nanocrystalline apatites revealed by dynamic nuclear polarization enhanced ^43^Ca NMR spectroscopy

**DOI:** 10.1038/ncomms14104

**Published:** 2017-01-27

**Authors:** Daniel Lee, César Leroy, Charlène Crevant, Laure Bonhomme-Coury, Florence Babonneau, Danielle Laurencin, Christian Bonhomme, Gaël De Paëpe

**Affiliations:** 1 Univ. Grenoble Alpes, INAC, F-38000 Grenoble, France; 2 CEA, INAC, F-38000 Grenoble, France; 3 Sorbonne Universités, UPMC Univ Paris 06, Collège de France, UMR CNRS 7574, Laboratoire de Chimie de la Matière Condensée de Paris, 4 place Jussieu, 75252 Paris cedex 05, France; 4 Institut Charles Gerhardt de Montpellier, UMR5253, CNRS UM ENSCM, CC1701, Place Eugène Bataillon, 34095 Montpellier cedex 05, France

## Abstract

The interfaces within bones, teeth and other hybrid biomaterials are of paramount importance but remain particularly difficult to characterize at the molecular level because both sensitive and selective techniques are mandatory. Here, it is demonstrated that unprecedented insights into calcium environments, for example the differentiation of surface and core species of hydroxyapatite nanoparticles, can be obtained using solid-state NMR, when combined with dynamic nuclear polarization. Although calcium represents an ideal NMR target here (and *de facto* for a large variety of calcium-derived materials), its stable NMR-active isotope, calcium-43, is a highly unreceptive probe. Using the sensitivity gains from dynamic nuclear polarization, not only could calcium-43 NMR spectra be obtained easily, but natural isotopic abundance 2D correlation experiments could be recorded for calcium-43 in short experimental time. This opens perspectives for the detailed study of interfaces in nanostructured materials of the highest biological interest as well as calcium-based nanosystems in general.

## Abstract (web summary)

Solid-state NMR can in principle be used to study calcium environments in biomaterials such as bones/teeth, but ^43^Ca lacks receptivity. Here the authors present an approach to acquire ^43^Ca data for hydroxyapatite at its natural isotopic abundance, distinguishing between core and surface Ca sites.

Calcium is an important element in many natural and synthetic materials. A key calcium-containing material is apatite, a calcium phosphate that is naturally present in sedimentary rocks in the form of fluorinated francolite, as well as in bones and teeth of vertebrae in the form of carbonated hydroxyapatite (Ca_10_(PO_4_)_6_(OH)_2_). The broad diversity of structural roles played by calcium compounds undoubtedly comes from the ability of Ca^2+^ cations to adopt a large variety of coordination environments, with coordination numbers varying from 4 to 9, and many different coordination geometries. Thus, determining the local environment of calcium is of fundamental importance to fully understand the structure and properties of these materials. Unfortunately, very few techniques are available to probe the local structure around calcium atoms, especially in disordered or amorphous materials, or in materials with several different calcium environments, as is the case in organic–inorganic biomaterials like bone, where calcium is present not only in the mineral phase but also at the organic–mineral interface. In a first rough approximation, nanosized synthetic carbonated hydroxyapatite (C–HAp), obtained by precipitation in aqueous medium, can act as a structural analogue for the inorganic component of bone tissues. Such nano-objects present intrinsic distributions of both cationic and anionic sites in the core as well as at the surface of the particles, leading to challenging analysis and interpretation.

Although solid-state nuclear magnetic resonance (NMR) has been used to study Ca^2+^ environments^1^,^2^,^3^,^4^,^5^, it has only rarely been applied to complex materials because of severe sensitivity drawbacks. Indeed, the natural abundance of ^43^Ca, the NMR-active stable isotope of calcium, is only 0.14%. Moreover, it has a small gyromagnetic ratio (|γ_Ca-43_|/γ_H-1_∼0.07) and spin-7/2, rendering it a highly insensitive nucleus. Thus, natural abundance experiments require large sample volumes (using 7 mm rotors or larger, limiting magic angle spinning (MAS) rates to ∼5 kHz) in combination with the highest magnetic field available and signal-enhancing NMR pulse sequences^2^,^5^,^6^,^7^,^8^,^9^,^10^, and even in these favourable conditions, experimental times for recording 1D ^43^Ca MAS NMR spectra can reach ∼7–10 h. It follows that attempting to gain further information from 2D experiments, recorded at natural abundance, remains unrealistic. In the case of ^43^Ca-labelled samples, ^43^Ca 2D and triple-resonance NMR experiments have been reported, showing that much greater insight into Ca^2+^ local environments can be accessed, but so far only a limited set of such experiments have been performed because the commercially-available ^43^Ca-labelled starting chemical, ^43^Ca-labelled calcite (CaCO_3_), is highly expensive^5^,^11^,^12^,^13^,^14^,^15^.

Recently, high-field dynamic nuclear polarization combined with magic angle spinning (MAS-DNP)^16^ has been shown to alleviate sensitivity limitations of NMR for both biological^17^ and material^18^ analytes and, accordingly, some of the difficulties when trying to observe low-gamma (|*γ*|≤|*γ*_N-15_|) nuclei^19^,^20^,^21^,^22^. This is because in MAS-DNP the large polarization of electron spins can be transferred to nuclei, greatly increasing their sensitivity in solid-state NMR experiments. Moreover, MAS-DNP experiments are generally performed with low sample temperatures of ∼100 K, which has been shown to be beneficial not only for the efficiency of the DNP process but also for cross-polarization (CP) of quadrupolar^23^ and low-gamma^20^ nuclei. Recent work using MAS-DNP has also shown its potential for studies of quadrupolar nuclei with low natural abundance, such as ^17^O (refs 24, 25). Nevertheless, fast ^43^Ca NMR at natural isotopic abundance still seemed a pipe dream owing to the intrinsic combination of NMR obstacles.

Herein, it is not only demonstrated that MAS-DNP can be used to facilitate the fast acquisition of ^43^Ca NMR spectra, but it is also shown that this can be done for samples at their natural isotopic abundance, even allowing the successful acquisition of 2D correlation experiments, which provide here the unambiguous discrimination of core and surface calcium sites in hydroxyapatite nanoparticles.

## Results

### The C–HAp nanoparticles

**Figure 1:**
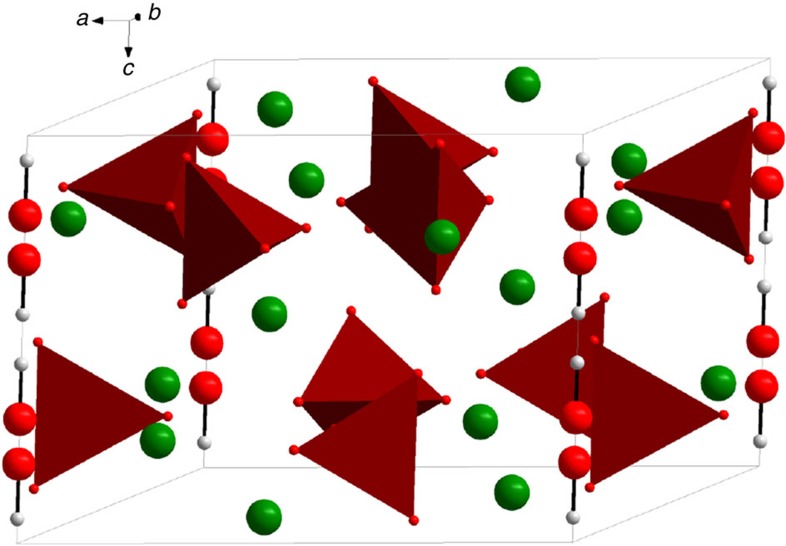
Representation of the unit cell of hexagonal HAp, Ca_10_(PO_4_)_6_(OH)_2_. Columns of OH^−^ (A-sites) are represented vertically (*c* axis) (Ca in green, O in red, H in white, PO_4_ tetrahedra in brown). ICSD-26204: Hollysprings hydroxyapatite, showing the orientational disorder along the OH columns.

The synthetic protocol to obtain the C–HAp nanoparticles is given in the ‘Methods' section and the routine characterization (transmission electron microscopy (TEM), X-ray powder diffraction (XRD), Fourier transform infrared spectroscopy (FTIR), thermal gravimetric analysis (TGA)) of the sample is presented in Supplementary Figs 1–4. Figure 1 displays a schematic representation of stoichiometric HAp. Carbonate groups can partially substitute OH^−^ (A-type) and/or PO_4_^3−^ moieties (B-type)^26^. The OH^−^ groups form columns (parallel to the *c* axis of the structure) that can be considered as rather isolated (the distance between two consecutive columns is ∼0.9 nm) and can act as paths for quasi one-dimensional ^1^H–^1^H spin-diffusion. It is worth noting that Klimavicius *et al*.^27^ demonstrated very recently that moderate MAS rates (up to 9 kHz) had little impact on the spin-diffusion in nanostructured HAp. Following the synthetic protocol, some Ca^2+^ cations were substituted by Na^+^ (see the chemical analysis in the Supplementary Methods).

### Preparation for DNP experiments

**Figure 2:**
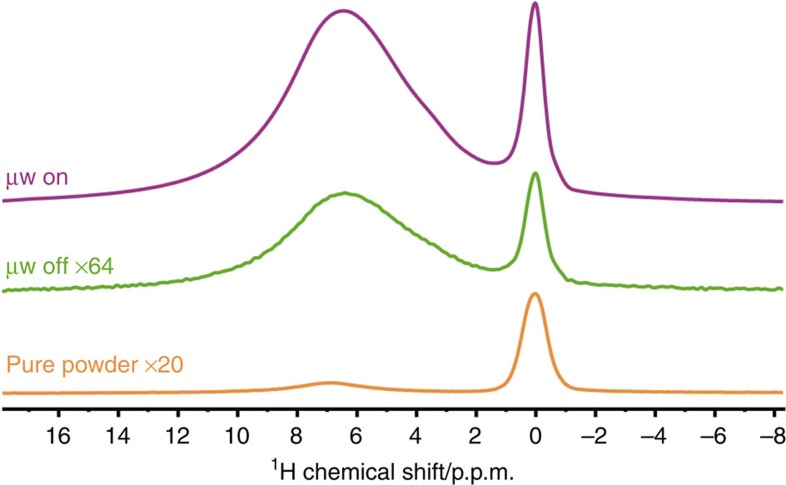
^1^H Hahn-echo MAS NMR spectra of C–HAp. All were recorded at ∼100 K and 9.4 T. The pure powder (orange, MAS rate=8.5 kHz) and the DNP-ready sample with (purple) and without (green) microwave irradiation suitable for DNP (both recorded using a MAS rate of ∼14 kHz) are shown and scaled according to the factors given.

Approximately 30 mg of the white nanoparticulate (diameter ∼30 nm, see Supplementary Fig. 1) C–HAp powder was wetted with a 30 μl aliquot of a glass-forming solvent matrix of glycerol-d_8_/D_2_O/H_2_O (60/30/10; v/v/v) containing 10 mM AMUPol^28^ biradical polarizing agent to create a DNP-ready sample^29^. The resulting slightly damp powder was packed into a 3.2 mm outer-diameter thin-wall zirconia MAS rotor and this was then inserted into the pre-cooled (100 K) MAS-DNP triple-resonance (^1^H/^13^C/^43^Ca) NMR probe and pneumatically spun to the desired rate using cold nitrogen gas. Under these MAS conditions, a few watts of microwave (μw) irradiation at approximately the Larmor frequency of the unpaired electron spins of AMUPol (∼263 GHz) are applied to the sample, inducing a transfer of the large spin polarization of the electrons to nearby nuclei^30^,^31^. For ^1^H nuclei, this is then equilibrated throughout the system via efficient ^1^H–^1^H spin-diffusion, resulting in a large net polarization gain among the ^1^H spins, as demonstrated in Fig. 2. Comparison with the signal returned in the absence of μw irradiation and also with the signal for a full rotor of the pure powder only (that is, no solvent matrix or polarizing agent), highlights the significant gain in signal intensity through the use of MAS-DNP. The absolute sensitivity ratio (ASR)^32^ is the best method to compare gains with MAS-DNP to conventional MAS NMR and gives a factor of ∼35 here for the OH^−^ groups, corresponding to an experimental time-saving of three orders of magnitude (35^2^). The OH^−^ groups are characterized by a sharp peak centred at *δ*_iso_(^1^H) ∼0 p.p.m., whereas a broad deshielded component assigned to hydrogen-bonded water/glycerol molecules is observed at *δ*_iso_(^1^H) ∼6.5 p.p.m. (independent experiments, not shown here, have demonstrated that HPO_4_^2−^ entities were almost entirely absent in the C–HAp sample). Note also the presence of a small, broad contribution at *δ*_iso_(^1^H) ∼6.8 p.p.m. for the pure powder, which arises from surface-absorbed water^33^.

To better understand the process by which the sample becomes hyperpolarized from the polarizing agents that are contained in the solvent, ^1^H NMR can be used here. The ^1^H spectra of the DNP-ready sample in Fig. 2 were obtained at a MAS rate of ∼14 kHz. The sharpening of the OH^−^ resonance compared with the spectrum recorded using a MAS rate of 8.5 kHz corroborates that ^1^H–^1^H spin-diffusion is still efficient under these moderate MAS rates as it implies the continued presence of residual ^1^H–^1^H dipolar couplings. This experimental fact is very important as it demonstrates that the ^1^H nuclei are still communicating along the OH^−^ columns. The communication is established via the residual ^1^H dipolar homonuclear interaction. The efficiency of this spin-diffusion process can be estimated more quantitatively: indeed, the ratio of signal intensities measured in the presence and absence of μw irradiation, *ɛ*_on/off_, is 120 for water/glycerol and 110 for C–HAp OH^−^ groups. Notably, these discrete but similar values can be measured since the OH^−^ resonance remains clearly distinguished from the solvent contribution. Such a similarity tends to prove that almost all protons belonging to the C–HAp nanoparticles are involved in the DNP process. Interestingly though, after saturation, the monoexponential magnetization build-ups for OH^−^ and the solvent (in the presence of biradicals) have time constants, *T*_B_(^1^H), that are quite different: 5 and 3 s, respectively. It follows that both ^1^H spin baths can be considered as quite disconnected. However, connections are observed between the OH^−^ and solvent resonances in a DNP-enhanced ^1^H–^1^H spin-diffusion correlation experiment, albeit only at relatively long spin-diffusion mixing times (Supplementary Fig. 5), indicating that the ^1^H spin baths do share some small connection. Hence, the hyperpolarization of ^1^H spins near to the polarizing agent can propagate from the solvent matrix into the nanoparticles via the OH^−^ channels (which are not large enough to accommodate the AMUPol molecules). In a second step, the ^1^H hyperpolarization can then be transferred to other nuclei of interest (here ^43^Ca (and ^13^C)), for example through a CP^34^ process.

### Studying Ca environments at natural abundance

**Figure 3:**
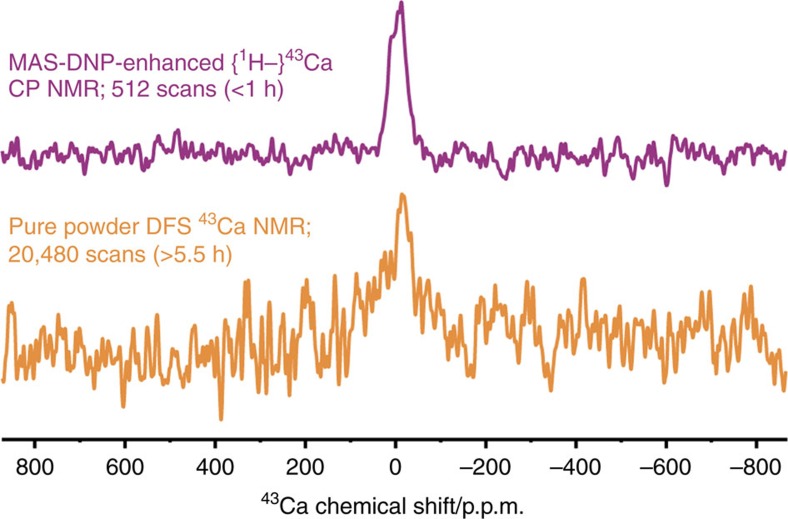
^43^Ca MAS NMR spectra of C–HAp. Both were recorded at ∼100 K, 9.4 T and using a MAS rate of 8.5 kHz. Direct CT excitation of ^43^Ca after DFS of the pure powder (orange) is compared with the DNP-enhanced {^1^H–}^43^Ca CP experiment (purple), recorded using a CP contact time of 5 ms. Note that the time required to obtain much greater S/N with the DNP-enhanced experiment was <1/5 compared with the conventional method; the experimental time-saving with the DNP-enhanced experiment is a factor of 15.

Many sensitivity enhancing experiments for solid-state NMR of quadrupolar nuclei have been devised^35^ and one of the most efficient involving population transfer, besides DNP, is a so-called double-frequency sweep (DFS)^36^ that can result in an increase of the population difference across the central transition (CT) by saturating the satellite transitions under MAS, giving NMR signal increases for the more readily observed CT of spin-7/2 nuclei of around a factor of 4 (for full satellite transition inversions a factor of 7 is attainable, at least theoretically)^15^. Figure 3 shows the ^43^Ca MAS NMR CT spectrum of C–HAp, recorded on the pure powder, after DFS, at a sample temperature of 100 K. Already by only using the low temperatures, the NMR signal-to-noise ratio (S/N) will be ∼3.6 times greater compared with conventional room temperature measurements^37^ as a result of the increased thermal nuclear polarization and decreased Johnson–Nyquist noise in the radio-frequency circuit of the NMR probe. This, combined with the DFS, will result in experiments requiring only one two-hundredth of the conventional acquisition time (time-saving=(3.6 × 4)^2^). Nevertheless, even with the low temperatures and the DFS, the aforementioned difficulties of ^43^Ca NMR cannot be surmounted and the S/N from the natural isotopic abundance sample is poor, even after 5.5 h of signal averaging. The long experimental times required to obtain sufficient S/N then render the acquisition of more complex experiments (such as 2D schemes) completely infeasible.

Figure 3 also shows the DNP-enhanced ^43^Ca MAS NMR spectrum of C–HAp, recorded using a ^1^H–^43^Ca CP step (that is, indirect DNP of ^43^Ca). Although CP to half-integer spin quadrupolar nuclei is usually very inefficient owing to the complex spin dynamics under MAS^38^, particularly for the case of ^43^Ca^5^, it is evident that the S/N is significantly better compared with the spectrum of the pure powder recorded with DFS, even with the much reduced experimental time (<1 h). It is worth highlighting that no data was recorded for the same experiment but in the absence of μw irradiation since this would have required months to obtain even a small signal. Therefore, the low efficiency of ^1^H–^43^Ca CP is completely negated by the large sensitivity enhancement from the MAS-DNP at low temperature, allowing fast acquisition of ^43^Ca NMR spectra at natural isotopic abundance.

There are two non-equivalent calcium sites, Ca(I) and Ca(II) (hexagonal symmetry, Ca(II)/Ca(I)=1.5, present in the structure of stoichiometric HAp (tentative fits of ^43^Ca NMR spectra based on previously published data^5^,^39^ are shown in Supplementary Fig. 6), with the Ca(II) sites being closer than Ca(I) sites to OH^−^ groups. The experimental spectra are compatible with the previously published data^5^,^39^, though slightly broadened. Such broadening has already been described experimentally at moderate field (8.45 T) and is assigned to distributions of the quadrupolar parameters^5^,^39^. At moderate fields, such distributions can play a significant role, especially in the case of nanoparticles for which surface and sub-surface defects are necessarily present in relatively high proportions. Furthermore, the substitution of some OH^−^/PO_4_^3−^ groups by carbonates and some Ca^2+^ by Na^+^ (see the chemical analysis in the Supplementary Methods) can contribute as another source of local disorder and thus also result in distributions of quadrupolar parameters and associated spectral broadening. Indeed, such broadening due to extended substitutions has been observed in natural samples such as teeth and bones^40^. It should also be noted that using CP can result in distorted quadrupolar lineshapes under MAS^41^.

### Interfaces in C–HAp nanoparticles

**Figure 4:**
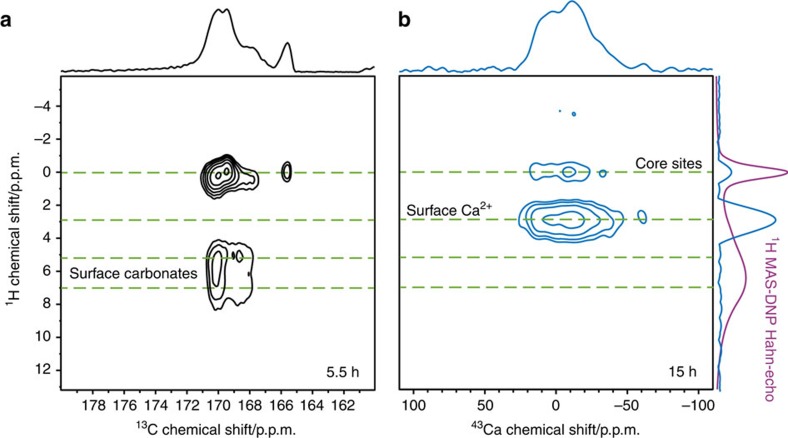
MAS-DNP HETCOR spectra of C–HAp. Both were recorded at ∼100 K, 9.4 T, and using a MAS rate of 8.5 kHz. (**a**) ^1^H–^13^C HETCOR spectrum with associated ^13^C skyline projection (top), recorded using a CP contact time of 7 ms. (**b**) ^1^H–^43^Ca HETCOR spectrum with associated ^43^Ca (top) and ^1^H (right) skyline projections, recorded using a CP contact time of 3 ms. Both spectra were recorded using FSLG homonuclear decoupling^48^ during the indirect acquisition and SPINAL-64 heteronuclear decoupling^49^ during the direct acquisition. Also shown (far right, purple) is the DNP-enhanced ^1^H Hahn-echo MAS NMR spectrum, for comparison.

As 1D DNP-enhanced {^1^H−}^43^Ca CP MAS NMR spectra could be recorded in a short experimental time for C–HAp, this experiment was easily extended to its 2D heteronuclear correlation (HETCOR) version^42^, which is used to probe proximities between the heteronuclei. A ^1^H−^43^Ca HETCOR spectrum of C–HAp is presented in Fig. 4b. To the best of our knowledge, this is the first ever obtained without any ^43^Ca labelling. Moreover, this was acquired in 15 h on a limited mass of sample (∼30 mg). For comparison, a 2D ^1^H-detected ^1^H–^43^Ca dipolar correlation spectrum of 30 mg 60% ^43^Ca-enriched HAp required ∼8 h of total experimental time at 14 T^14^, meaning that ∼150 days would be needed for a natural abundance (0.14%) sample. Most interestingly, two correlation peaks are clearly distinguished using a short CP contact time of 3 ms (in contrast with experiments at longer contact times of 7 ms, in which only the one correlation peak at *δ*_iso_(^1^H) ∼0 p.p.m. is present—see Supplementary Fig. 7). As expected, ^43^Ca sites are correlated to the columnar OH^−^ groups at *δ*_iso_(^1^H) ∼0 p.p.m. A strong correlation is also evidenced at *δ*_iso_(^1^H) ∼3 p.p.m., although a ^1^H resonance related to this chemical shift can only be observed as a shoulder in Fig. 2. The global linewidth of the ^1^H projection centred at *δ*_iso_(^1^H) ∼3 p.p.m. is estimated to ∼2.7 p.p.m. (from 1.7 to 4.4 p.p.m.). Such a range of ^1^H chemical shifts is compatible with those of hydrogen-bonded hydroxyls^43^, indicating an assignment to surface OH^−^ groups that are hydrogen-bonded to surface-bound water or surface-adsorbed solvent molecules. Contributions from pure solvent protons can be ruled out because these are much more deshielded, at *δ*_iso_(^1^H) ∼6.5 p.p.m. as shown in Fig. 2. This is corroborated by the ^1^H−^13^C HETCOR spectrum of C–HAp, which shows correlations for the ^13^C resonances only with ^1^H species with *δ*_iso_(^1^H)=6.3 p.p.m. (FWHM ∼3 p.p.m.) (Supplementary Fig. 7a). Since the DNP solvent mixture (glycerol-d_8_/D_2_O/H_2_O (60/30/10; v/v/v)) was prepared at room temperature there will most likely be deuterium/proton equilibration of the exchangeable groups, resulting in 1.8 times more OH groups from water compared with glycerol, with both of these proton environments then contributing to this correlation at *δ*_iso_(^1^H)=6.3 p.p.m. Furthermore, it should be noted that polyols such as glycerol (and carbohydrates as well) can chelate Ca^2+^ cations^44^,^45^,^46^,^47^. So it cannot be ruled out that there is a ^1^H−^43^Ca dipolar interaction between residual (non-hydrogen-bonded) glycerol molecules from the DNP solvent that are chelated to surface Ca^2+^ sites, leading to a correlation at *δ*_iso_(^1^H) ∼3 p.p.m. Therefore, it follows that this correlation is then fully attributable to ^1^H nuclei at the surface (that is, not in the columnar channels) of the C–HAp nanoparticles, either from OH^−^ groups or chelating glycerol molecules, that are coupled to surface or near-surface Ca^2+^ cations.

At longer ^1^H−^43^Ca CP contact times spin decoherence effects will become more important, reducing the efficiency of CP for local spin systems with larger ^1^H–^1^H dipolar couplings and those that are in closer proximity to the electron spins from the polarizing agent. Hence, CP for the isolated OH^−^ groups of the columns (*vide supra*) should be less affected than for the surface OH^−^ groups (or solvent molecules), which is indeed the case here (see the ^1^H−^43^Ca HETCOR spectrum of Supplementary Fig. 7b where a CP contact time of 7 ms was used, showing only a dominant correlation peak at *δ*_iso_(^1^H) ∼0 p.p.m.). The correlation centred at *δ*_iso_(^1^H) ∼0 p.p.m. in Fig. 4b is assigned to ^43^Ca nuclei located near the hydroxyl columns, that is, in the core of the nanoparticles. A comparison of the ^43^Ca cross-sections taken through the surface and core correlations shows that the surface related ^43^Ca spectrum is slightly broader (Supplementary Fig. 8). This could be expected because of structural disorder at the surface, leading to additional distributions of chemical shift and/or quadrupolar parameters.

The ^1^H–^13^C HETCOR spectrum of C–HAp is presented in Fig. 4a. It is clearly demonstrated that even in the case of ^13^C at natural abundance (1.1%) and with only ∼1 wt% in carbon, 2D DNP-enhanced ^1^H–^13^C HETCOR spectra can be obtained in short experimental time (here 5.5 h). One notes also that ^13^C resolution remains almost identical to that observed at room temperature. Following Beshah *et al*.^48^, the most shielded ^13^C resonance (*δ*_iso_(^13^C)=166 p.p.m.) can be assigned to A-site carbonates whereas all deshielded resonances correspond to B-site carbonates. Looking carefully at the correlations in the 2D ^1^H–^13^C HETCOR experiment provides more detailed information. (i) As expected, ^13^C resonances are coupled to channel OH^−^ protons, corresponding then to core carbonates. (ii) Some B-type resonances are correlated to the most deshielded part of the ^1^H spectrum, corresponding then to surface carbonates. There seems to be two discrete ^1^H environments associated to these surface carbonates: at *δ*_iso_(^1^H) ∼7 p.p.m. and *δ*_iso_(^1^H) ∼5 p.p.m., most likely from distinct surface OH groups. Notably, the proton correlations related to these surface carbonates are clearly distinguished from the one related to the surface Ca^2+^ cations. In other words, the multinuclear MAS-DNP experiments are selective towards the different protonated species present at the surface of the C–HAp nanoparticles. (iii) The A-site resonance (*δ*_iso_(^13^C)=166 p.p.m.) is mainly correlated to the OH^−^ groups in the *c* axis columns. (iv) The lack of any ^13^C correlations with a peak at *δ*_iso_(^1^H) ∼3 p.p.m. (using CP contact times of 0.25, 1 and 7 ms (only the latter shown here)) allows the refinement of the previous assignment (*vide supra*). This peak must then be specifically from (hydrogen-bonded) calcium-bound hydroxyls on the surface of the nanoparticles, or from calcium-chelated glycerol molecules (which have a lesser affinity to carbonates).

The ability to selectively probe either the surface or the core of materials based on HAp at natural isotopic abundance is important, particularly so for the former. Indeed, the structure of tissues like bone and teeth is still the matter of much debate^49^,^50^,^51^,^52^. Many recent studies show that there is a real need to develop new ways of analysing these materials at different length-scales, and notably at the molecular level^53^, to determine how the different individual components (proteins, mineral, water molecules and so on) are associated together, and how this can have an impact on the functional properties of the tissue. In this context, the organic–mineral interface has drawn much attention, revealing the importance of water molecules, small molecular anions like citrate, and non-collagenous proteins like osteopontin and osteocalcin, though detailed insight on their mode of binding to bone mineral is still missing. Given that these species are expected to coordinate to the surface Ca^2+^ sites (through ionic interactions and/or coordination bonds), directly analysing the surface Ca^2+^ sites of HAp in native tissue would be a way to directly investigate this mode of binding. The natural isotopic abundance MAS-DNP experiments proposed here illustrate that such experiments are now feasible, which clearly opens avenues for understanding the structure of the mineral interface in these important materials. A preliminary attempt was made to record a DNP-enhanced {^1^H−}^43^Ca CP MAS NMR spectrum of mice teeth (Supplementary Fig. 9). The observation of a ^43^Ca NMR signal shows the applicability of the experiment to natural samples, although further investigation into the sample preparation and acquisition conditions is required to optimize the MAS-DNP experiments.

It has been demonstrated that C–HAp nanoparticles can be studied efficiently and effectively using MAS-DNP. Although ^43^Ca should be a fundamental NMR target in the field of biomaterials and biominerals, it suffers from severe drawbacks in terms of intrinsic sensitivity (low *γ*, high spin number and very low natural abundance). Nevertheless, ^1^H−^43^Ca 2D experiments were recorded at natural isotopic abundance in short experimental time, notably for a restricted mass of sample (∼30 mg). To date, such experiments would have been impossible to implement using conventional NMR. An analysis of the ^1^H−^43^Ca and ^1^H−^13^C correlation data showed that surface species (Ca^2+^ and carbonates) could be distinguished from the core entities. Such results open avenues for the detailed atomic-scale description of interfaces in bones and teeth, which is of primary importance to understand their structure and properties^53^, as well as for calcium-derived nanomaterials in general.

## Methods

### Carbonate substituted hydroxyapatite (C–HAp)

The protocol used corresponded to the standard precipitation of HAp at room temperature starting from aqueous solutions (initially decarbonated by boiling the solutions and using an argon flux during the syntheses). A solution of ammonium hydrogenphosphate ((NH_4_)_2_HPO_4_, 0.30 mol l^−1^, pH ∼10) was added to a solution of calcium nitrate tetrahydrate (Ca(NO_3_)_2_.4H_2_O, 0.50 mol l^−1^, pH ∼5) using a titration apparatus (808 Titrando, Methrom, 3 ml min^−1^). Carbonates were introduced by adding sodium hydrogencarbonate (NaHCO_3_) to the phosphate solution. All experiments were performed under argon flux and magnetic stirring. The initial molar ratios were: Ca:10, P:6, CO_3_^2−^:3. After the addition of the phosphate solution, the obtained precipitates were further stirred for 24 h. After centrifugation, the precipitates were rinsed four times with distilled water (final pH of water ∼7). All samples were then heat treated (400 °C) for 48 h to eliminate water and ammonia molecules. Routine characterization (TEM, XRD, FTIR, TGA) of the sample is presented in Supplementary Figs 1–4.

### Solid-state NMR experiments

The glycerol-d_8_ (98% in D atoms) and D_2_O (> 99% in D atoms) used for the glass-forming solvent matrix were standard commercial reagents and the AMUPol^28^ biradical polarizing agent was purchased through SATT Sud Est, Marseille, France. Solid-state NMR experiments were recorded using a Bruker DNP-NMR AVANCE III 400 MHz spectrometer equipped with a gyrotron and associated transmission line capable of delivering ∼5 W of ∼264 GHz μw irradiation at the sample. All experiments were recorded with a 3.2 mm HXY triple-resonance MAS probe (in *λ*/4 ^1^H transmission mode) at *ν*_0_(^1^H)=400.33 MHz corresponding to the maximum ^1^H enhancement field position for AMUPol at *ν*_0_(e^−^)=263.7 GHz, with the X-channel tuned to *ν*_0_(^13^C)=100.66 MHz and the Y-channel tuned to *ν*_0_(^43^Ca)=26.94 MHz. A home-adapted triple-resonance ^1^H/^13^C/^43^Ca circuit was constructed using a 15 pF capacitor in parallel to an insert coil of seven turns, used to bridge the X and Y channels, and also a 64 pF shunt capacitance in parallel to the variable Y capacitor. The Y-channel tuning range at low temperature (∼100 K) using this setup is 26.9±0.8 MHz.

^1^H (*π*/2)- and *π*-pulses, as well as the (SPINAL-64 (ref. 54)) heteronuclear decoupling, were applied to induce a nutation frequency of 100 kHz. ^13^C CP spin-locking was at ∼50 kHz with corresponding ramped (50–100%) ^1^H CP spin-locking at ∼76 kHz (100%) to meet a CP-matching condition and facilitate CP transfer. The ^1^H spin-lock during ^1^H–^43^Ca CP transfer was applied in a low nutation frequency regime (∼12 kHz) to permit relatively efficient CT-selective CP of ^43^Ca nuclei, for which a (*n*=1, zero quantum) CP-matching condition was met using ^43^Ca radio-frequency irradiation at ∼0.9 kHz. A long dead time of 120 μs was used to avoid the acquisition of ^43^Ca signals resulting from probe ringing. For ^13^C acquisition, this dead time was only 7 μs. The {^1^H–}^43^Ca CP and ^43^Ca direct excitation after DFS preparation were calibrated using a (60%) ^43^Ca-labelled sample of Mg-substituted HAp, which was synthesized using a similar protocol to that described previously^11^. For the ^43^Ca NMR spectra shown in Fig. 3, the recycle delays were 6.5 s for the MAS-DNP {^1^H–}^43^Ca CP NMR experiment (value directly optimized on the sample) and 1 s for the DFS–^43^Ca NMR experiment (the ^43^Ca relaxation of precipitated hydroxyapatite is known to be fast compared with other Ca-containing materials^55^).

### Data availability

The data that support the findings of this study are available from the corresponding author upon reasonable request.

## Additional information

**How to cite this article:** Lee, D. *et al*. Interfacial Ca^2+^ environments in nanocrystalline apatites revealed by dynamic nuclear polarization enhanced ^43^Ca NMR spectroscopy. *Nat. Commun.* **8,** 14104 doi: 10.1038/ncomms14104 (2017).

**Publisher's note:** Springer Nature remains neutral with regard to jurisdictional claims in published maps and institutional affiliations.

## Supplementary Material

Supplementary Information Supplementary Figures, Supplementary Methods and Supplementary References.
