# Supplementary material for: Interfacial Ca2+ environments in nanocrystalline apatites revealed by dynamic nuclear polarization enhanced 43Ca NMR spectroscopy
Source: Nat Commun. 2017 Jan 27;8:14104. doi: 10.1038/ncomms14104 (PMC5290151; doi:10.1038/ncomms14104)
Supplement: Supplementary Information — Supplementary Figures, Supplementary Methods and Supplementary References. [file ncomms14104-s1.pdf]

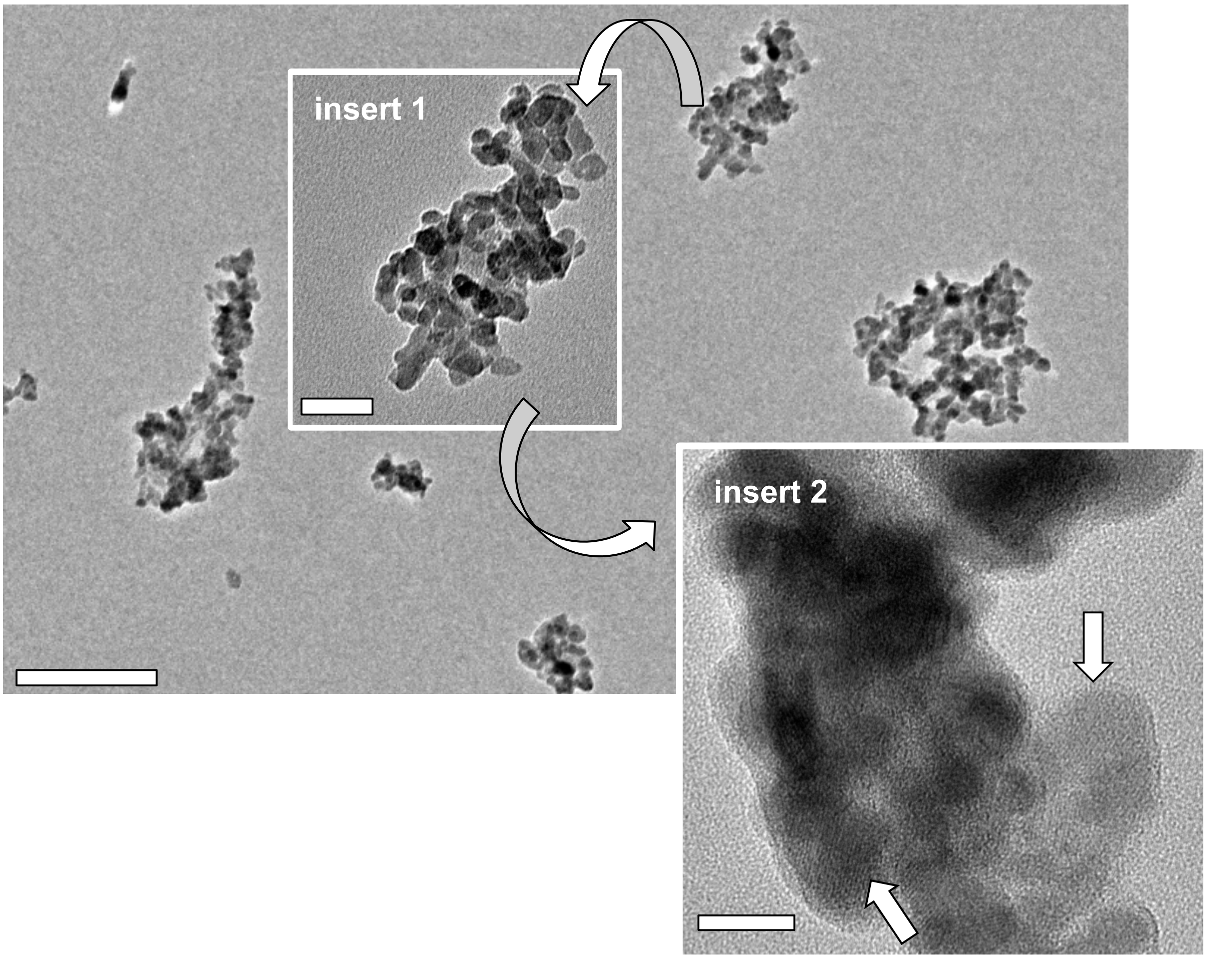

Supplementary Figure 1. Transmission Electron Microscopy (FEI TECMAI G2 Spirit Twin electron microscope, 120 kV): Aggregates of nanoparticles (spheroidal shape) were evidenced; scale bar, 200 nm. Insert 1: an average size of the spheroids is ~ 30 nm; scale bar, 50 nm. Insert 2: diffraction planes were clearly evidenced; scale bar, 20 nm.

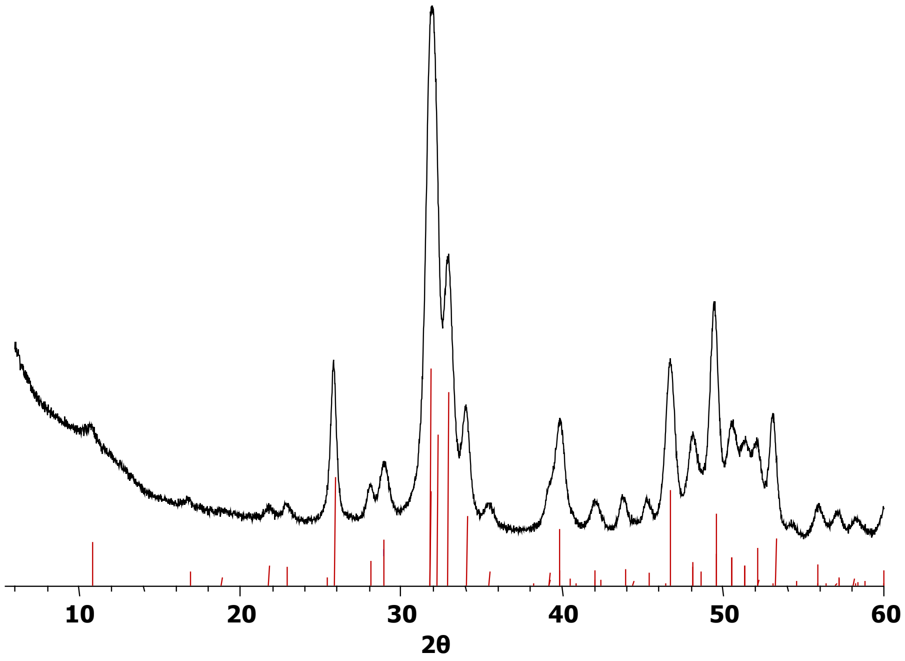

Supplementary Figure 2. Powder XRD (Bruker D8 ADVANCE, scan mode 2θ = 4°-60°, Cu Kα, λ = 1.5406 Å). All peaks were safely assigned to hexagonal hydroxyapatite phase (ICSD-26204, data in red). The broadening of the diffraction peaks can be attributed to the nanometric size of the particles and to partial distribution by carbonates.

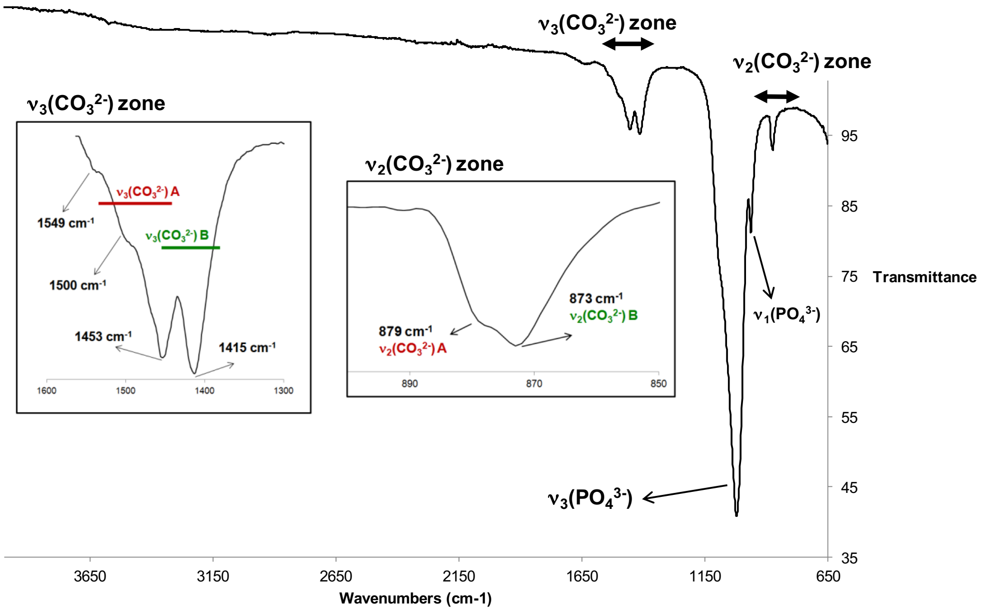

Supplementary Figure 3. FTIR spectroscopy (Perkin-Elmer spectrum 400 F-IR/FT-NIR spectrometer, universal ATR sampling accessory, 4500-600 cm^{-1}). Bands located at 1549, 1500, 1453, 1415, 879 and 873 cm^{-1} are characteristic for carbonates in A (OH^-) and B (PO_4^{3-}) sites. Following Fleet[1], the FTIR spectrum corresponds to a Na^+ substituted AB C-HAp with both A- and B-type substitutions[2]. The presence of Na^+ in the apatitic structure is clearly demonstrated by the shift of the $\nu_3(CO_3^{2-})$ (asymmetric stretch) doublet for A-type carbonates into the region normally associated with B-type carbonates. Consequently, a very strong overlap between FTIR bands is observed (see the $\nu_3(CO_3^{2-})$ zone). The region at ~ 875 cm^{-1} is associated to $\nu_2(CO_3^{2-})$ (out-of-plane bend) (see the $\nu_2(CO_3^{2-})$ zone).

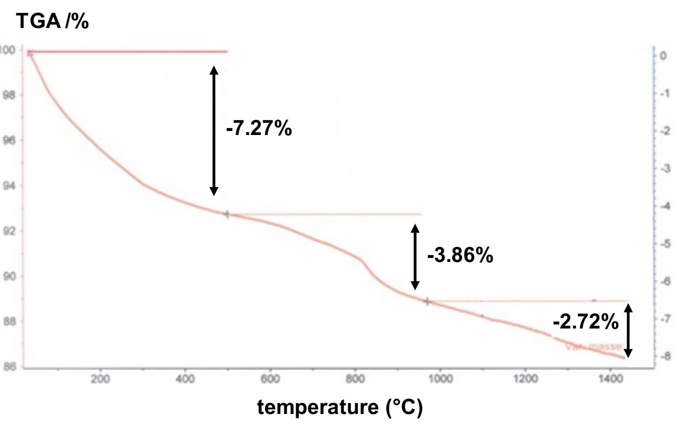

Supplementary Figure 4. TGA analyses (Netzsch STA409PC TGA/DTA apparatus). 30 to 400°C: loss of water and ammonia. 400 to 1000°C: loss of carbonates. T > 1000°C: loss of water by dehydroxylation.

(a)

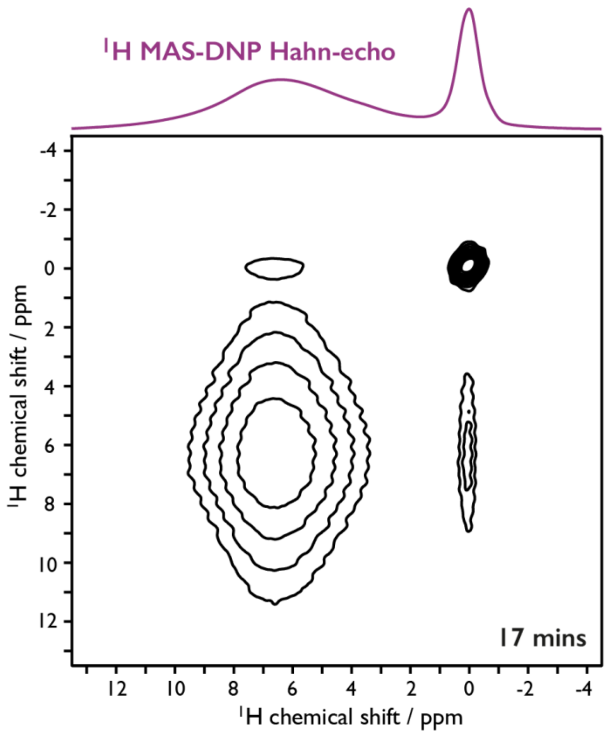

(b)

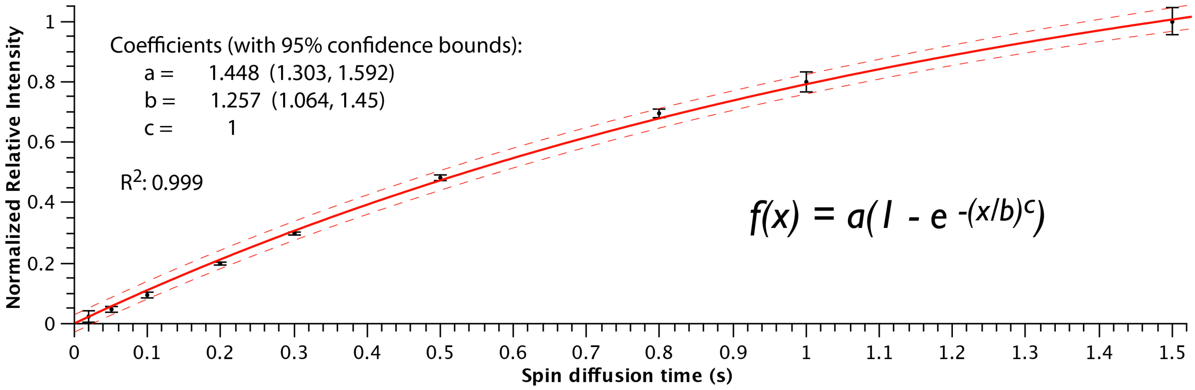

Supplementary Figure 5. DNP-enhanced ^{1}H-^{1}H spin-diffusion experiment[3] data for C–HAp, recorded at ~ 100 K, 9.4 T, and using a MAS rate of 8.5 kHz. (**a**) 2D experiment recorded using a mixing time of 300 ms, illustrating the presence of cross-peaks. Also shown (top, purple) is the DNP-enhanced ^{1}H Hahn-echo MAS NMR spectrum, for comparison. (**b**) Relative intensity for the cross-peak at 0 ppm in the ω_2 dimension as a function of the spin-diffusion mixing time. The error bars highlight the errors associated with the S/N of the cross-peak. The solid red line is the fit to the given equation with the given coefficients and the dashed red lines are the 95 % confidence bounds of the fit.

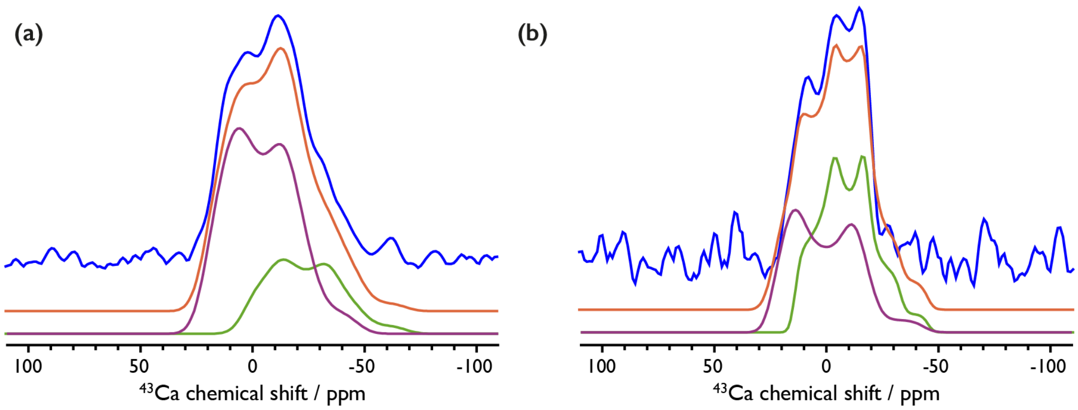

Supplementary Figure 6. DNP-enhanced {^{1}H-}^{43}Ca CP MAS NMR. In blue: experimental spectra taken from skyline projections from the corresponding HETCOR spectra recorded with CP contact times of 3 ms (**a**) and 7 ms (**b**). In red: spectral fitting using the NMR parameters of Ca(I) (green lineshape): (**a**) $\delta_{iso}(^{43}Ca) = 7 \pm 7$ ppm, $C_Q = 2.6 \pm 0.4$ MHz, $\eta_Q = 0.4 \pm 0.2$, (**b**) $\delta_{iso}(^{43}Ca) = 11 \pm 9$ ppm, $C_Q = 2.6 \pm 0.4$ MHz, $\eta_Q = 0.4 \pm 0.3$; and Ca(II) (purple lineshape): (**a**) $\delta_{iso}(^{43}Ca) = 25 \pm 5$ ppm, $C_Q = 2.6 \pm 0.4$ MHz, $\eta_Q = 0.3 \pm 0.2$, (**b**) $\delta_{iso}(^{43}Ca) = 25 \pm 2$ ppm, $C_Q = 2.6 \pm 0.2$ MHz, $\eta_Q = 0.4 \pm 0.1$. These are similar to those published in the literature for HAp[4]. It needs to be highlighted that these are tentative fits, and are used only for illustrative purposes. Further experiments[4] would be required to properly determine the NMR parameters of each Ca site, such as multiple magnetic field analyses and/or MQMAS experiments. Particularly, the intensities of the fits depend on the CP conditions so they cannot be considered as quantitative. Nevertheless, it is not possible to fit this data with only one Ca site, and also it is clear that the Ca signals are significantly broader in (**a**) than (**b**).

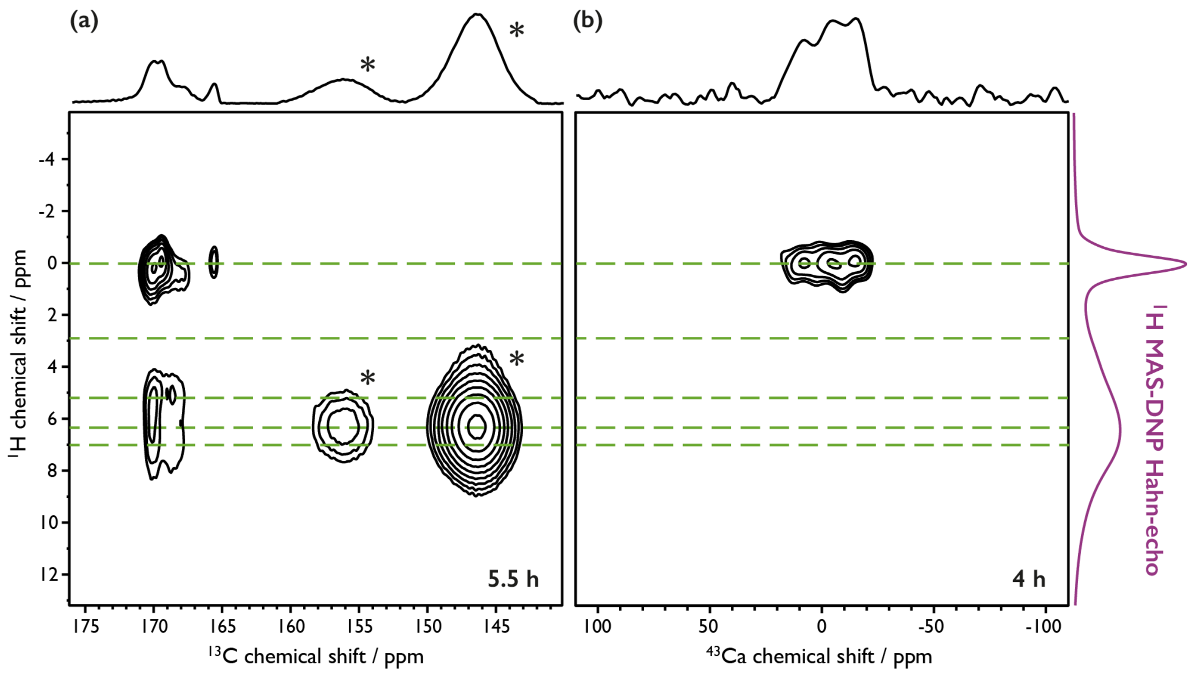

Supplementary Figure 7. MAS-DNP heteronuclear correlation (HETCOR) spectra of C–HAp with associated skyline projections (top), recorded at ~100 K, 9.4 T, using a MAS rate of 8.5 kHz, and CP contact times of 7 ms. From the ^{1}H-^{13}C correlations (**a**), it is evident that carbonate in the C–HAp is close to apatite hydroxyls as well as water/glycerol ^{1}H nuclei. At the longer contact times used here for the ^{1}H-^{43}Ca correlations (**b**) (7 ms, *cf*. the 3 ms used for Figure 4b in the main manuscript) the apatite hydroxyl correlations dominate and no peak at $\delta_{iso}(^1H)$ ~ 3 ppm is observed. Peaks denoted with * are spinning sidebands from the glycerol peaks (observed at $\delta_{iso}(^{13}C)$ = 62 and 71 ppm). Also shown (far right, purple) is the DNP-enhanced ^{1}H Hahn-echo MAS NMR spectrum, for comparison.

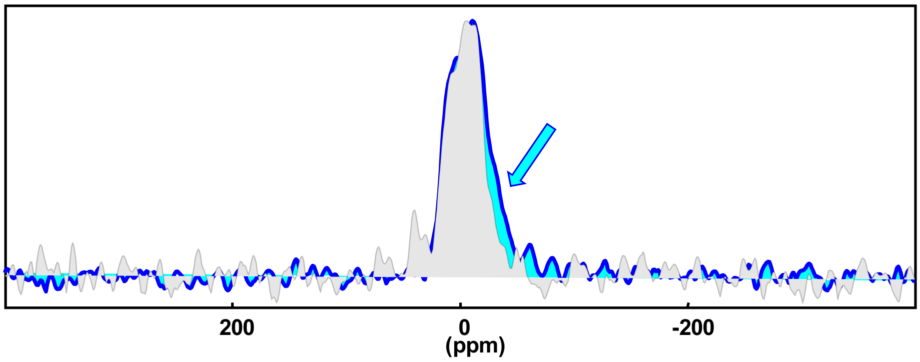

Supplementary Figure 8. Cross-sections through DNP-enhanced ^{1}H-^{43}Ca HETCOR NMR spectra. In blue: ^{43}Ca cross-section of the signal related to $\delta_{iso}(^{1}H)$ ~ 3 ppm (CP contact time: 3 ms, from Figure 4b in the main manuscript). In grey: ^{43}Ca cross-section of the signal related to $\delta_{iso}(^{1}H)$ ~ 0 ppm (CP contact time: 7 ms, from Supplementary Figure 7). The blue arrow highlights the larger linewidth of the blue component.

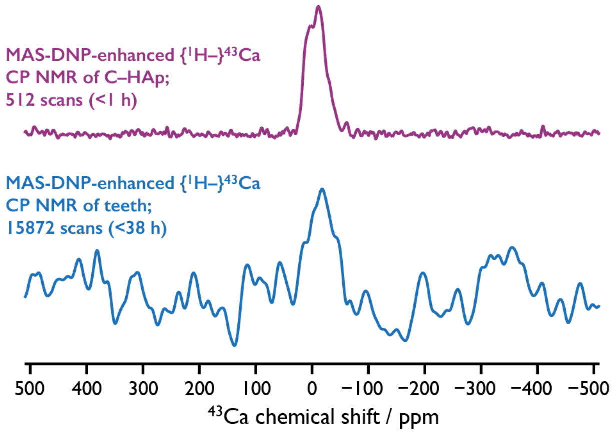

Supplementary Figure 9. DNP-enhanced {^{1}H-}^{43}Ca CP MAS NMR spectra of mice teeth (blue, bottom) and C–HAp (purple, top, taken from Figure 3 in the main manuscript and shown as a comparison), recorded at ~100 K, 9.4 T, using a MAS rate of 8.5 kHz, and CP contact times of 5 and 6 ms for the C–HAp and mice teeth, respectively. The mice teeth were extracted from euthanized mice and stored at -80 ºC. Prior to the experiment, they were thawed, washed once in H_2O_2, and then subsequently with H_2O. In preparation for the MAS-DNP experiments, the treated mice teeth were then ground by hand gently at room temperature, before being wetted with glycerol-d_8/D_2O/H_2O (60/30/10; v/v/v) containing 10 mM AMUPol biradical polarizing agent.

Supplementary Methods

Chemical analysis: performed at the Centre d'Analyses CNRS, Vernaison, France. Found wt%: Ca:34.17, P:15.66, C:1.04, Na:0.33, N:1.23. $Ca_{10}Na_{0.17}P_{5.93}C_{1.02}N_{1.03}O_zH_w$. CO_3^{2-} wt%: 5.20.

Supplementary References

(1) *Carbonated Hydroxyapatite: Materials, Synthesis, and Applications*, Fleet, M. E., Ed. (Pan Stanford Publishing Pte Ltd, 2015).

(2) Fleet, M. E. & Liu, X. *Biomaterials* **28,** 916–926 (2007).

(3) Jeener, J., Meier, B. H., Bachmann, P. & Ernst, R. R. *J. Chem. Phys.* **71,** 4546 (1979).

(4) Laurencin, D., Wong, A., Dupree, R. & Smith, M. E. *Magn. Reson. Chem.* **46,** 347–350 (2008).
